# A novel mutation in *RNF216* gene in a Turkish case with Gordon Holmes syndrome

**DOI:** 10.1186/s12920-023-01529-4

**Published:** 2023-05-09

**Authors:** Nazlı Durmaz Çelik, Ebru Erzurumluoğlu, Serkan Özben, Uğur Toprak, Göknur Yorulmaz, Sevilhan Artan, Serhat Özkan

**Affiliations:** 1 Department of Neurology, Eskişehir Osmangazi University Faculty of Medicine, Eskişehir, Turkey; 2 Department of Medical Genetics, Faculty of Medicine, Eskişehir Osmangazi University, Eskişehir, Turkey; 3 Department of Neurology, University of Health Sciences, Antalya Training and Research Hospital, Antalya, Turkey; 4 Department of Radiology, Eskişehir Osmangazi University Faculty of Medicine, Eskişehir, Turkey; 5 Department of Endocrinology, Eskişehir Osmangazi University Faculty of Medicine, Eskişehir, Turkey

**Keywords:** Gordon Holmes syndrome, Hypogonadotropic hypogonadism, Cerebellar ataxia, *RNF216*

## Abstract

**Background:**

Gordon Holmes syndrome (GHS) is a rare autosomal recessive disorder characterized by hypogonadotropic hypogonadism, cognitive decline, and cerebellar ataxia. Mutations in the Ring Finger Protein 216 (*RNF216*) gene have been known to be associated with GHS therewithal *RNF216* mutations have been detected in cases with Huntington-like disease, 4H syndrome (hypodontia, hypomyelination, ataxia and hypogonadotropic hypogonadism), and congenital hypogonadotropic hypogonadism.

**Case presentation:**

Here we report a novel homozygous frameshift mutation in *RNF216* gene c.1860_1861dupCT (p.Cys621SerfsTer56) in a patient with hypogonadotropic hypogonadism, ataxia, and cognitive decline diagnosed with GHS also co-occurrence of parkinsonism and dystonia which was not reported before.

**Conclusions:**

We report an extremely rare case of GHS. The core features of GHS are well defined, but genotype–phenotype correlations are still limited. To understand the pathophysiology of different phenotypes, the type and localization of novel mutations need to be defined, and the effect of these different variants on clinical features needs to be determined. Further studies should explain the factors of phenotypic variability present in GHS patients with *RNF216* mutations.

**Supplementary Information:**

The online version contains supplementary material available at 10.1186/s12920-023-01529-4.

## Background

Gordon Holmes syndrome (GHS) (MIM #212840) is a rare autosomal recessive neurodegenerative disorder. It was first described by British neurologist Holmes in 1907 [1] and characterized by hypogonadotropic hypogonadism, cognitive decline, and cerebellar ataxia. Recently, mutations in Ring Finger Protein 216 (*RNF216*), *OTUD4, STUB1* and *PNPLA6* genes were reported to be associated with GHS [2]. *RNF216* and *PNPLA6* are the most frequently mutated genes in GHS. *RNF216* encodes the E3 ubiquitin-protein ligase that is responsible for regulation of autophagy and regulates synaptic transmission and plasticity in neurons [3, 4]. In addition to GHS, *RNF216* mutations have been detected in cases with Huntington-like disease (HLD), 4H syndrome (hypodontia, hypomyelination, ataxia and hypogonadotropic hypogonadism), and congenital hypogonadotropic hypogonadism (HH) [5–7].

Here we report a novel homozygous frameshift mutation in *RNF216* gene c.1860_1861dupCT (p.Cys621SerfsTer56) in a patient with hypogonadotropic hypogonadism, ataxia, dystonia, and cognitive decline diagnosed with GHS.

## Case presentation

The proband (IV:4) is a-23-year-old male with a four-year history of difficulty in walking and frequent falls. He also has clumsiness in both his arms and hands and complains about speech difficulty for about the last six months. The patient was the product of consanguineous parents and he was born by successful vaginal delivery with normal birth parameters. His mental and psychomotor history revealed that he left school at the age of 14 due to learning difficulties. In his medical history, at age 18, he was found to have gynecomastia and small testicles in his routine examination before military compulsory service.

### Physical and neurologic examination

Physical examination revealed eunuchoid body proportions, short stature, gynecomastia, and poor facial hair growth with generalized jaundice appearance. His neurological examination showed dysarthria, and severe ataxia making his walking impossible without assistance. He had appendicular dysmetria and dysdiadochokinesia, especially in both lower extremities, slightly generalized chorea while talking, hypomimia, mild bradykinesia, slight dystonia in the left hand, brisk deep tendon reflexes in lower extremities. Eye examination showed fragmented pursuit eye movements with slow hypometric saccades, vertical gaze palsy, and square wave jerks in horizontal pursuit (Additional file 1: Video 1). In the psychiatric examination, he had regressed speech, and looked small compared to his peers, there was no delirium suicide, no homicidal thoughts, and no euthymic perception deviation. His IQ test reported borderline mental capacity (Table 1). His Kent EGY intelligence test verbal performance was 85.71. He couldn't get a calculable score from the50 Porteus maze test.

**Table 1:** Summary of the clinical, neuroimaging, and genetic features of Gordon Holmes patients with RNF216 mutations (Adopted from Gonzales- Latapi et al. [2] and Wu et al. [10]

Family and Patient (Author)	Sex	Age of onset (years)	Clinical type	Clinical feature	Pubertal development	Imaging findings	RNF216 genotype (NM_ 207,111.3)
F1-P1 (Margolin DH et al.)	M	22	GHS	Dysarthria, ataxia, dementia, died at 43 yr	No puberty	Cerebellar and cerebral atrophy, cerebral WMLs	c.2251C > T(p.R751C); c.2251C > T(p.R751C)
F1-P2 (Margolin DH et al.)	F	20	GHS	Personality change, dysarthria, ataxia, dementia, died at 41 yr	Normal puberty, secondary amenorrhea	Cerebellar and cerebral atrophy, cerebral WMLs	c.2251C > T(p.R751C); c.2251C > T(p.R751C)
F1-P3 (Margolin DH et al.)	M	29	GHS	Dysarthria, ataxia and dementia, died at 47 yr	Normal puberty, erectile dysfunction	Cerebellar and cerebral atrophy, cerebral WMLs	c.2251C > T(p.R751C); c.2251C > T(p.R751C)
F2-P4 (Margolin DH et al.)	M	22	GHS	Dysarthria, ataxia, dementia, chorea,gaze-evoked nystagmus, died at 36 yr	No puberty	Cerebellar atrophy, WMLs surrounding the basal ganglia, hyperintensities in basal ganglia, thalami and midbrain	c.615_616delGA(p.E205DfsX15);c.1791 T > A(p.C597X)
F3-P5 (Margolin DH et al.)	F	27	GHS	Ataxia, dysarthria, dementia	No puberty	Multiple foci of subcortical WMLs, cerebellar atrophy	c.721C > T( p.Q241X)
F4-P6 (Margolin DH et al.)	M	21	GHS	Slurred speech, ataxia, mood changes, memory impairment	No puberty	Cerebellar atrophy, cerebral atrophy, foci of WMLs	c.2149C > T(p.R717C)
F5-P7 (Alqwaifly M et al.)	M	20	GHS	Mild ataxia	Poor development of puberty	Mild cerebellar atrophy, subcortical WMLs	c.2061G > A(splicing); c.2061G > A(splicing)
F5-P8 (Alqwaifly M et al.)	M	24	GHS	Ataxia, dementia, dysarthria, broken saccadic eye movement, exaggerated deep tendon reflexes	Poor development of puberty	Cerebellar atrophy, subcortical WMLs	c.2061G > A(splicing); c.2061G > A(splicing)
F6-P9 (Mahmood S, et al.)	M	22	GHS	Limb and gait ataxia, slurred speech, subcortical dementia, chorea, died at 35 yr	No puberty	Cerebellar atrophy, WMLs in both cerebral hemispheres, grey matter lesions in the thalami, T2 hyperintensities in basal ganglia, thalami, and midbrain	c.615_616delG(p.E205fsX15); c.1791 T > A(p.C597X)
F7-P10 (Calandra CR et al.)	M	28	GHS	Ataxia, dysarthria, brisk tendon reflexes, dementia	Poor development of puberty	Cerebral WMLs, cortical and cerebellar atrophy	c.1988C > T(p.P663L); c.1988C > T(p.P663L)
F7-P11 (Calandra CR et al.)	M	27	GHS	Dysarthria, ataxia, brisk reflexes, cognitive impairment	Poor development of puberty	Cerebral WMLs, cortical and cerebellar atrophy	c.1988C > T(p.P663L); c.1988C > T(p.P663L)
F8-P12 (Chen et al.)	M	33	GHS	Dysarthria, ataxia, slurred speech, cognitive impairment	Post pubertal infertility	Cerebral WMLs, cerebellar atrophy	c.1948G > T (p.E650X)
F9-P13 (Wu CJ et al.)	M	26	GHS	Dysarthria, ataxia, cognitive decline	No puberty	Cerebellar and cerebral atrophy, supratentorial WMLs, involvement of brainstem and thalami	c.1549C > T(p.R517X); c.1549C > T(p.R517X)
F 10-P14 Çelik et al.	M	18	GHS	Dysarthria, severe ataxia, appendicular dysmetria, and dysdiadochokinesia, slightly generalized chorea parkinsonizm, slight dystonia, fragmented pursuit eye movements, slow hypometric saccades, vertical gaze palsy, and square wave jerks in horizontal pursuit	No puberty	Severe cerebellar and vermis atrophy, dilated third and lateral ventricles, slight cerebral cortical atrophy, mesencephalic slight atrophy, and periventricular confluent white matter lesions	c.1860_1861dupCT (p.Cys621SerfsTer56)

*GHS* Gordon Holmes Syndrome, *WMLs* white matter lesions

### Imaging

**Fig. 1:**
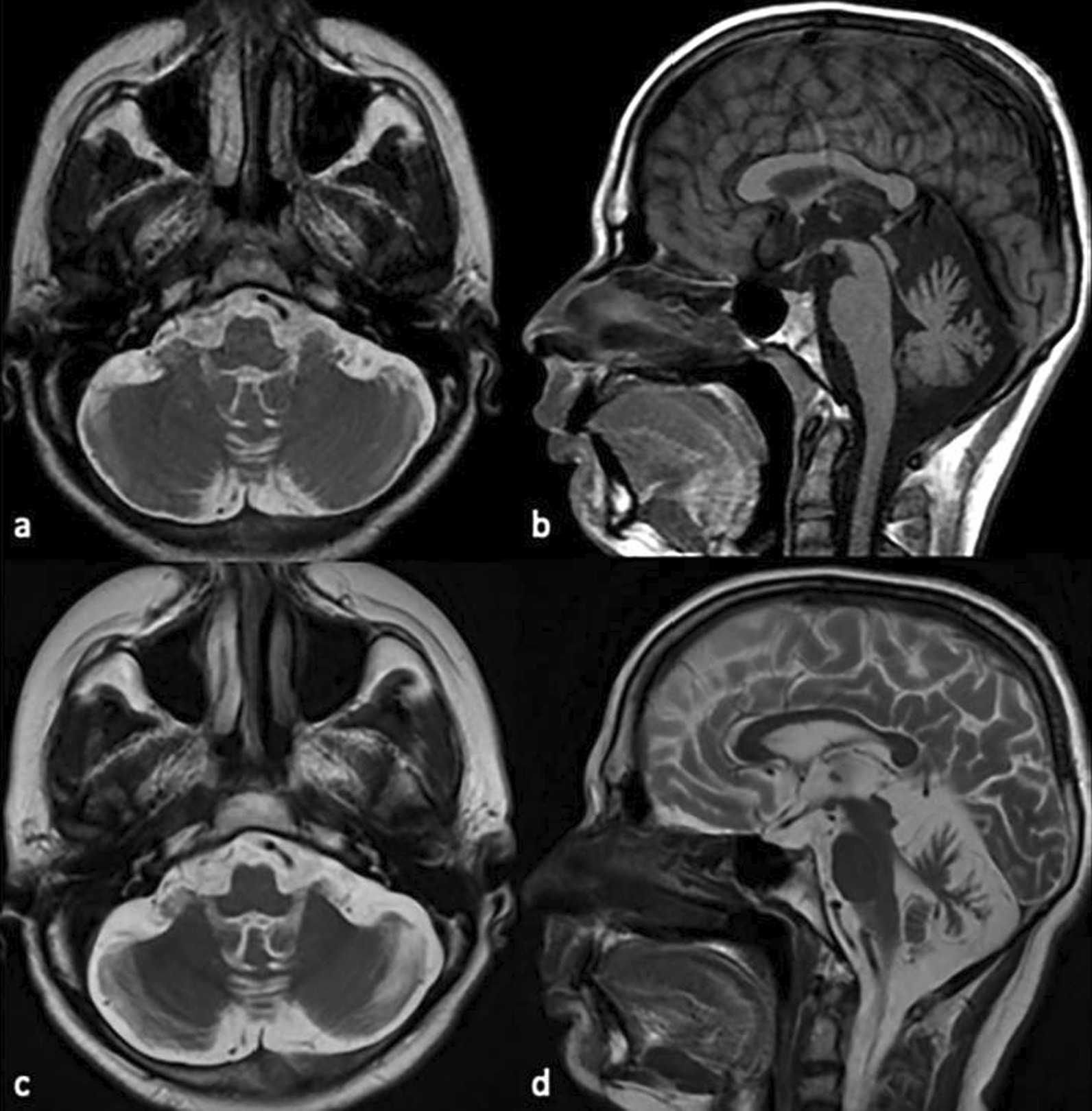
MRI of the case in 2018 and 2022. According to 2018 (**a**, axial T2 and **b**, sagittal T1-weighted) MRI in 2022 (**c** axial, **d** sagittal T2-weighted) progression is seen in cerebellar, vermian and cerebral cortical atrophy. Mild atrophy in the mesencephalon has not progressed

**Fig. 2:**
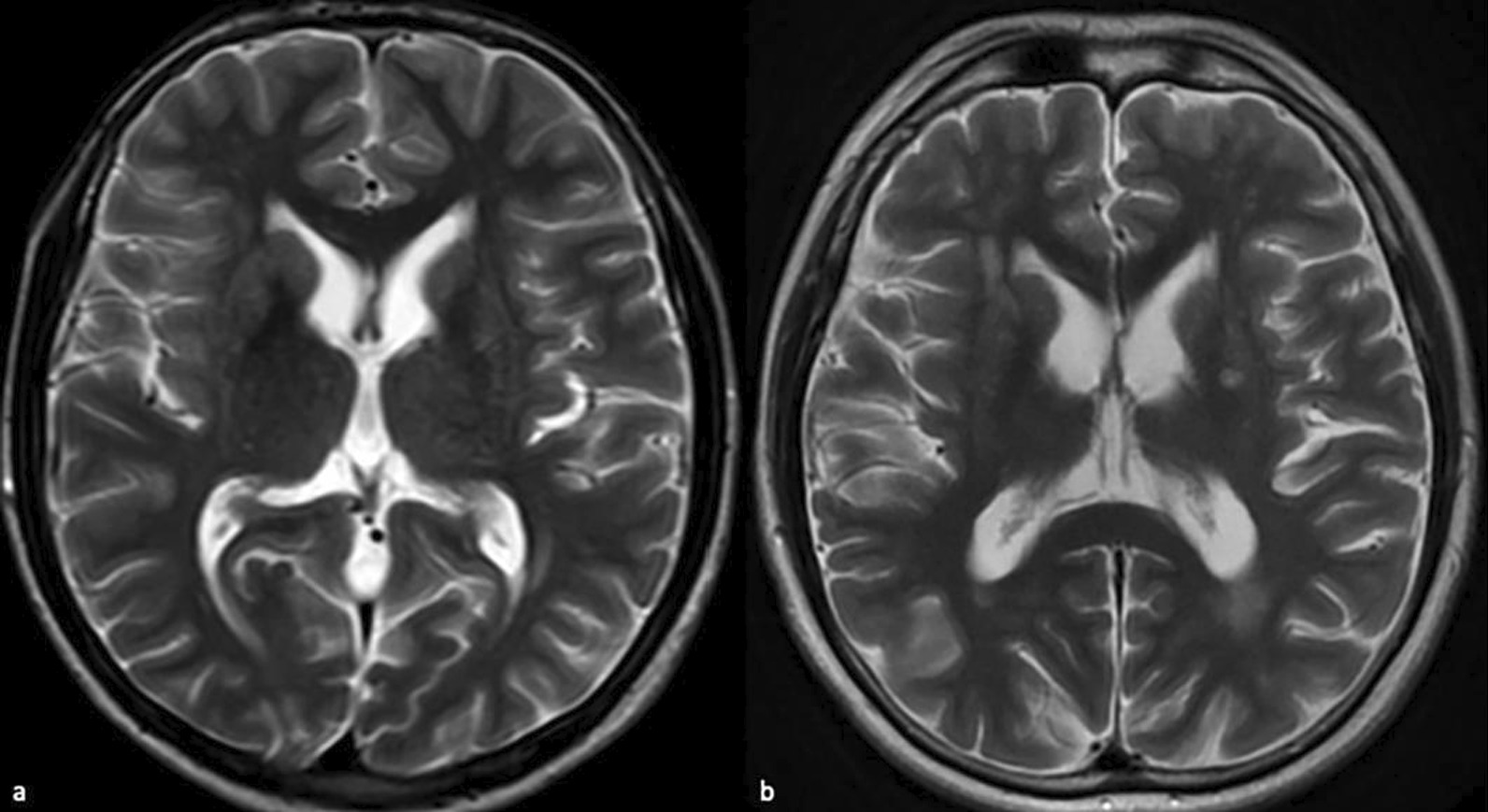
MRIs of the case in 2018 (**a**) and 2022 (**b**). Axial T2-weighted images show progression in periventricular confluent hyperintensities and cerebral cortical atrophy. Basal ganglia hyperintensities appear in 2022

Brain magnetic resonance imaging (MRI) revealed progressive cerebellar, vermian, and cerebral cortical atrophy, and periventricular confluent white matter hyperintensities in 2022 compared to 2018. Mild mesencephalic atrophy is similar in both dates (Fig. 1). Basal ganglia hyperintense lesions began to appear in 2022 (Fig. 2).

Pituitary MRI showed a normal pituitary gland height (4 mm) for his age. Testicular atrophy detected on ultrasound (right testis 12 × 6 × 15mm, 0.56 ml, left 10 × 4.5 × 14 mm, 0.32 ml). In the X-ray evaluation of his hands at 24, it was noted that the growth plate of the distal radius, which normally starts at the age of 17 to 19 and should fuse at the most at the age of 20, did not fuse.

### Laboratory

Laboratory findings including total blood count, renal and liver functions, thyroid hormone, thyroid antibodies, and vitamin E levels were normal. His blood glucose level [121 mg/dl (70–110)], HbA1c [5.8% (3.5–5.6)], and serum lipid levels (cholesterol [216 mg/dl (118–199)], LDL [156.9 mg /dl (66–129)] were slightly high. His HDL level [34 mg/dl (40–63)] was slightly low, and triglyceride level [121 mg/dl (44–149)] was normal. His basal hormonal evaluation was normal, but his follicle-stimulating (FSH) and luteinizing hormone (LH) levels [FSH < 0.3 (1.5–12.4 mIU/mL), LH: < 0.3 (1.7–8.6 mIU/mL)], respectively), testosteron level [0.44 nmol/L (9.9–27.8 nmol)], and free androgen index 2.75% (14.8–94.8) were low.

His growth hormone was higher than normal [4.18 ng/ml (0.030–2.47)], which was consistent with HH. The patient described here consistently presented with ataxia, cognitive deterioration, and HH, leading to the clinical diagnosis of GHS.

### Molecular analyses

Genomic DNA was isolated from peripheral blood using QIAamp DNA Blood Mini Kit), according to the recommendations of the manufacturer. Genetics analyses were performed by next-generation sequencing (NGS) using a Custom Target Capture Neuromuscular NGS Panel, consisting of 293 genes (Additional file 2) related to genetic neuromuscular diseases (Celemics, Korea). Variant calling and analysis were performed using “SEQ” variant analysis software (Genomize, Istanbul, Turkey) according to the reference genome of GRCh37 (hg19). Variants with a minor allele frequency (MAF) higher than 0.1% in Genome Aggregation Database (http://gnomad.broadinstitute.org/) were filtered out. We interpreted the identified variants using The Human Gene Mutation Database (http://www.hgmd.cf.ac.uk/ac/), ClinVar (http://www.ncbi.nlm.nih.gov/clinvar/), and literature search. Variants were classified according to the American College of Medical Genetics and Genomics guidelines for the interpretation of sequence variants (ACMG) [8]. Segregation analyses and variant validation were performed by direct sequencing using capillary electrophoresis (3130xl Genetic Analyzer, Applied Biosystems).

## Genetic analysis results

**Fig. 3:**
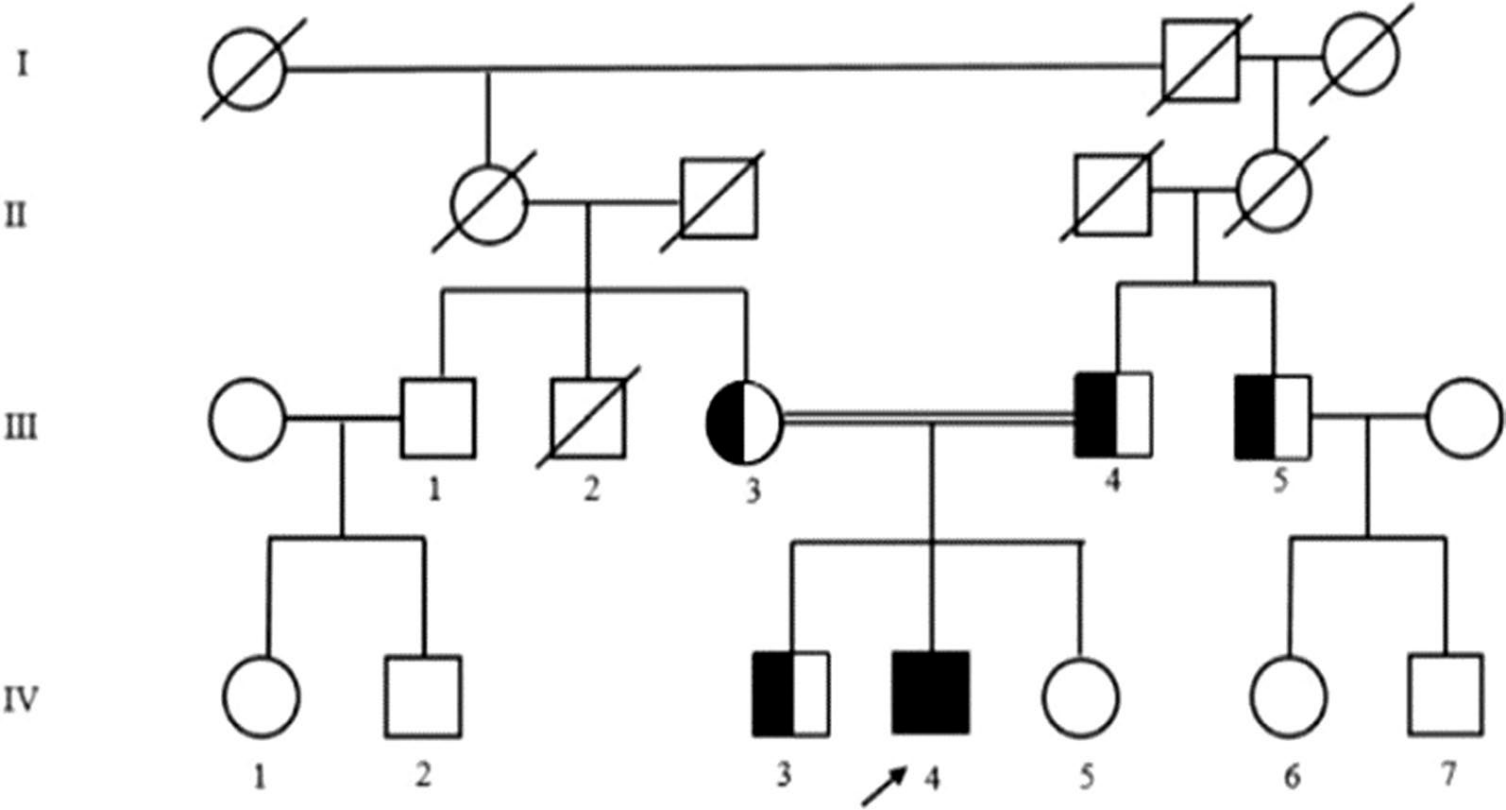
Pedigree of the family. The index case is marked with the arrow

The next-generation sequencing analyses of the proband (IV:4) identified a novel homozygous frameshift mutation (ENST00000389902.3):c.1860_1861dupCT (p.Cys621SerfsTer56) in exon 12 of the *RNF216* gene. This novel variant is predicted to result in a truncated protein by forming a premature stop codon and was classified as pathogenic along with PVS1 (null variant), PM2 (absent from controls (gnomAD, 1000 Genomes Project) and highly conserved position), PP3 (pathogenic computational predictions), PP4 (Patient’s phenotype is highly specific for a disease with a single genetic etiology) according to American College of Medical Genetics and Genomics guidelines. The mutation with heterozygous state was carried by consanguineous parents (III:3, III:4). The unaffected brother (IV:3) and uncle of the proband (III:5) had the same frameshift mutation with heterozygous state (Fig. 3).

## Discussion and conclusions

Here, we report a novel homozygous *RNF216* p.Cys621SerfsTer56 mutation in a Turkish patient presenting with Gordon Holmes syndrome.

*RNF216* gene encodes the E3 ubiquitin-protein ligase that is responsible for the regulation of autophagy and also regulates synaptic transmission and plasticity in neurons [3] Loss-of-function mutations in the *RNF216* gene are related to pathological effects on the cerebellum, hippocampus, cerebral white matter, hypothalamus, and pituitary components of the reproductive endocrine cascade [9]. So far, *RNF216* mutations have been detected in 13 patients with GHS in nine families [5, 9–12]. Additionally, *RNF216* mutations have also been identified in patients diagnosed with HLD, 4H syndrome, and congenital HH [6, 7, 9, 13–15]. Hitherto, the most common clinical features detected in cases with GHS are cognitive decline, ataxia, dysarthria, and poor pubertal development. In our patient, severe ataxia, cognitive deterioration, and dysarthria were also found to be consistent with the literature. The presence of parkinsonism, dystonia, and chorea differs our patient from previous cases (Table 1). Although chorea has been reported as a common symptom of *RNF216*-related HDL, it was found in only one case diagnosed with *RNF216*-related GHS. Notably, both cases with GHS chorea had a frameshift variant and a relatively early age of onset [9]. Also inactivating mutations in PNPLA6, STUB1, and OTUD4 have also been identified in GHS which are also ubiquination-related genes. PNPLA6 (19p13.2, (patatin-like phospholipase domain containing 6) encodes neuropathy target esterase, a phospholipid deacetylase converting phosphatidylcholine into fatty acids and glycerophosphocholine. *STUB1* ((16p13.3,STIP1 homology and U-box containing protein 1) encodes CHIP which is a key component of general cellular protein homeostasis, which, as RNF216, acts as a ubiquitin ligase. *OTUD4* (4q31.21 OTU domain-containing protein 4) encodes deubiquitinase OTUD 4 hich hydrolyzes the isopeptide bond between the ubiquitin C-terminus and the lysine epsilon-amino group of the target protein. The phenotype of GHC cases caused by these genes are summarized in Table 2 [2, 11].

**Table 2:** Summary of the clinical, neuroimaging, and genetic features of Gordon Holmes patients with PNPLA6, STUB1 and OTUD4 genes mutations (Adopted from Gonzales- Latapi et al. [2] and Wu et al. [11]

Family and Patient (Author)	Sex	Age of onset (years)	Clinical type	Clinical feature	Pubertal development	Imaging findings	Genotype and mutation
F1-P1 (Margolin, DH. et al.)	M	22	GHS	Ataxia, dementia	No puberty	Cerebellar and cerebral atrophy, cerebral WMLs	*OTUD4* pG333V and *RNF216* p.R751C
F1-P2 (Margolin, DH. et al.)	F	16	GHS	Personality change, ataxia, dementia,	Secondary amenorrhea	Cerebellar and cortical atrophy, hrpoerintensitieas in cerebral white matter	*OTUD4* pG333V and *RNF216* p.R751C
F1-P3 (Margolin, DH. et al.)	M	29	GHS	Ataxia, dementia,	Normal puberty Erectile dysfunction	Cerebral and diffuse cortical atrophy, multiple punctate and confluent areas of hyperintensity	*OTUD4* pG333V and *RNF216* p.R751C
F2-P2 (Shi, C. et al.)	F	17	GHS	Gait ataxia Appendicular ataxia Cerebellar ocular abnormalities Hand tremor and coarse head tremor Pyramidal signs Cognitive impairment	Hypogonadotropic Primary amenorrhea Underdeveloped secondary sexual characters Hypoplasia of uterus and ovaries	Cerebellar atrophy	*STUB1* homozygous mutation (c. 737C > T)
F2-P3 (Shi, C. et al.)	F	19	GHS	Gait ataxia Appendicular ataxia Cerebellar ocular abnormalities Cognitive impairment	Hypogonadotropic Primary amenorrhea Underdeveloped secondary sexual characters Hypoplasia of uterus and ovaries	Cerebellar atrophy	*STUB1* homozygous mutation (c. 737C > T)
F3-P4 Hayer SN, et al.,	M	12	GHS	Ataxia, spasticity, bilateral Babinski’s sign, focal dystonia, hypomimia, severe cognitibe impairment, urge incontinence Epilepsy	hypogonadism	Cerebellar, mesencephalic and parieto-occipital cortical atrophy	*STUB!* c.880A > T (p.Arg119*) c.880A > T (p.Ile294Phe)
F3-P5 Hayer SN, et al.,	M	12	GHS	Ataxia, spasticity, urge incontinence, intermittent ballistic athetotic movements, epilepsy, severe cognitive impairment, mutism	normal	Cerebellar atrophy	*STUB!* c.433A > C (p.Lys145Gln)
F3-P6 Hayer SN, et al.,	F	20	GHS	Spasticity, intermittent ballistic athetotic movements, epilepsy,severe cognitive impatiment, mutism	n/a	Severe cerebellar atrophy, vermis and hemispheric atrophy	*STUB!* c.433A > C (p.Lys145Gln)
F4-P7 (Synofzik, M. et al.)	M	6	GHS	Dysarthria, ataxia, brisk tendon reflexes	Delayed puberty	Cerebellar atrophy, empty sella	PNPLA6 c.3084_3085insGCCA p.Arg1031Glufs*38 c.4084C 4G p.Arg1362Gly
F 5-P8 (Teive, H. et al.)	M	23	GHS	Ataxia	Poor development of puberty	Mild cerebellar atrophy	*PNPLA6* c.4081C > T, p.Arg1381* c.3373G > A, p.Asp1125Asn
F6- P9 (Locci, S. et al.)	M	25		Spastic ve ataxic gait, central nystagmus in the lateral gaze, slight dysmetria, dysdiadochokinesia, milf pyramidal hypertonia, bilateral ankle clonus, brisk deep tendon reflexes	Poor development of puberty	Cerebellat atrophy, small bilateral hyperintense lesions in the periventricular white matter	*PNPLA6* c.2264 A > C; (p.Gln755Pro) c 0.3388 C > T; (p.His1130Tryr)
F7- P10 (Salgado, P. et al.)	F	25	GHS	Gait ataxia, mild dysarthria, cognitive decline, horizontal and vertical gaze-evoked nystagmus, postural hand tremor	No puberty	Cerebellar and vermis atrophy	*PNPLA6* compound heterozygosity [c.2404G > C]; p.(Glu802Gln); [c.4081C > T],p.(Arg1361*)
F7- P11 (Salgado, P. et al.)	F	N/R	GHS	Cognitive decline	No puberty	Cerebellar atrophy	*PNPLA6* compound heterozygosity [c.2404G > C]; p.(Glu802Gln); [c.4081C > T],p.(Arg1361*)
F7- P12 (Salgado, P. et al.)	F	N/R	GHS	Ataxia and tremor, cognitive decline	No puberty	Cerebellar atrophy	*PNPLA6* compound heterozygosity [c.2404G > C]; p.(Glu802Gln); [c.4081C > T],p.(Arg1361*)
F7- P13 (Salgado, P. et al.)	F	N/R	GHS	Ataxia and tremor, epilepsy and migraine, cognitive decline	No puberty	Cerebellar atrophy	*PNPLA6* compound heterozygosity [c.2404G > C]; p.(Glu802Gln); [c.4081C > T],p.(Arg1361*)
F8-P14 Topaloğlu, H., et al	M	37		Gait ataxia, dysartria	Poor development of puberty	Cerebellar atrrophy	*PNPLA6* c.3380C > G; p.S1127C
F8- P15 Topaloğlu, H., et al	F	20 s		Gait ataxia	Hypogonadotrophic hypogonadism	Cerebellar atrrophy	*PNPLA6* c.3380C > G; p.S1127C
F8-P16 Topaloğlu, H., et al	M	N/R	21	Gait ataxia	Hypogonadotrophic hypogonadism	Cerebellar atrrophy	*PNPLA6* c.3380C > G; p.S1127C
F8-P17 Topaloğlu, H., et al	M		18	Nystagmus, gait ataxia, dysartira	N puberty	Cerebellar atrophy	*PNPLA6* c.3380C > G; p.S1127C
F8-P18 Topaloğlu, H., et al	M		15	N/R	No puberty	N	*PNPLA6* c.3380C > G; p.S1127C

*F *female, *GHS* Gordon Holmes Syndrome, *M* male, *N/R* not reported,*WMLs* white matter lesions

The age of onset of neurological symptoms in GHS was observed at the beginning of the third decade in the cases reported so far. Our patient's first complaint was at the age of 18, and it is the youngest age of symptom onset reported. Brain MRI showed extensive middle and subcortical confluent white matter lesions, cerebral and cerebellar atrophy, which are consistent with the other previous cases. There were also T2 hyperintense areas consistent with putaminal degeneration, which is not common in GHS. Basal ganglia hyperintense lesions were reported by Margolin et al.‘s patient presenting with chorea mentioned before, reported to be associated with *RNF216* frameshift mutation. In a recent study, white matter lesions surrounding the basal ganglia were associated with only chorea compared to all *RNF216* mutated patients and their imaging findings so far [11], this finding is also compatible with the MRI findings of our case (Fig. 1). Chorea and parkinsonism developed after other symptoms in our patient, and the appearance of hyperintense lesions in the basal ganglia on MRI four years later is consistent with these findings. Neurocognitive assessment batteries were performed in the previous cases, but our patient could not cooperate with the neurocognitive batteries, so cognitive evaluation was performed with IQ tests. Hypogonadotropic hypogonadism is a common feature of GHS and has been demonstrated in all *RNF216* mutations, including our case. Our patient is being followed up with testosterone isocaproatetherapy.

## Conclusion

Here we present a case with Gordon Holmes syndrome caused by a novel *RNF216* mutation. This syndrome is very rare, and it has been recently found to be associated with the *RNF216* mutation. Ataxia, cognitive decline, and hypogonadotropic hypogonadism are the core features of this syndrome, but despite thorough literature research, we did not identify a paper that reported co-occurrence of parkinsonism and dystonia with other features in GHS.

Genotype–phenotype correlations are still limited. To understand the pathophysiology of different phenotypes, the type and localization of novel mutations need to be defined, and the effect of these different variants on clinical features needs to be determined.

## Supplementary Information

**Additional file 1**. The neurologic evamination of the patient. **Segment 1.** Physical examination: revealed eunuchoid body proportions, short stature, gynecomastia, and poor facial hair growth with generalized jaundice appearance, Speech and movements of eyes: The patient is asked “How old are you?” “Do you have a sibling?” “Where do you live?” “Do your brother or sister have a similar disease?” “did you goto school by the examiner. Speech initiation is delayed and speech production is slowed and dysarthric. He is hypomimic. Eye examination showed fragmented pursuit eye movements with slow hypometric saccades, vertical gaze palsy, and square wave jerks in horizontal pursuit. slightly generalized chorea while talking. **Segment 2.** Movements of the extremities: Movements in the patient revealed slight dystonia in the left hand and head, dystonic tremor while moving his arms. He also has appendicular dysmetria. **Segment 3.** Walking: The patient has severe ataxia making his walking impossible without assistance.

**Additional file 2**. The gene list of neuromuscular Next-Generation sequencing panel.

## Data Availability

The datasets generated and/or analysed during the current study has been submitted to the "Global Variome shared LOVD" and that can be accessed using 'https://databases.lovd.nl/shared/individuals/00430226.

## Abbreviations

GHS: Gordon Holmes syndrome
*RNF216*: Ring finger protein 216
MRI: Magnetic resonance imaging
HLD: Huntington-like disease
HH: Hypogonadotropic hypogonadism
NGS: Next-generation sequencing
