# Supplementary material for: A novel mutation in RNF216 gene in a Turkish case with Gordon Holmes syndrome
Source: BMC Med Genomics. 2023 May 9;16:98. doi: 10.1186/s12920-023-01529-4 (PMC10169457; doi:10.1186/s12920-023-01529-4)
Supplement: Supplementary file 2 — Additional file 2. The gene list of neuromuscular Next-Generation sequencing panel. [file 12920_2023_1529_MOESM2_ESM.docx]

**Supplementary material.**

**Celemix Neuromuscular kit**

AARS, ABCB7, ABCD1, ABHD12, ACAD9, ACADL,ACADM, ACO2, ACTA1, ADCK3, AFG3L2, AGL, AIFM1,ALDH3A2, AMPD1, ANO10, ANO5, AP4B1, AP4E1,AP4M1, AP4S1, AP5Z1, APTX, ARSA, ATCAY, a, ATM,ATP2A1, ATP7A, ATP7B, ATP8A2, BAG3, BEAN1, BIN1,BSCL2, C10orf2, C12orf65, C19orf12, CACNA1A,CACNA1S, CACNB4, CAPN3, CASK, CAV3, CCDC78,CCDC88C, CFL2, CHAT, CHRNA1, CHRNB1, CHRND,CHRNE, CHRNG, CLCN1, CLCN2, CLN5, CNTN1,COL6A1, COL6A2, COL6A3, COLQ, CPT1B, CPT2,CRYAB, CTDP1, CWF19L1, CYP27A1, CYP2U1, CYP7B1,DAG1, DCTN1, DDHD1, DDHD2, DES, DMD, DNAJB2,DNAJB6, DNM2, DNMT1, DOK7, DYNC1H1, DYSF,EEF2, EGR2, ELOVL4, ELOVL5, EMD, ERLIN2, ETFA,ETFB, FA2H, FAM134B, FGD4, FGF14, FHL1, FIG4,FKRP, FKTN, FLNC, FLVCR1, FRMD7, FUS, FXN, GAA,GAD1, GALC, GAN, GARS, GBA2, GDAP1, GJB1, GJC2,GLA, GLE1, GNB4, GNE, GOSR2, GPR143, GRID2,GRM1, GYS1, HADHA, HADHB, HINT1, HOXD10,HSPB1, HSPB8, HSPD1, HSPG2, IGHMBP2, IKBKAP,ISPD, ITGA7, ITPR1, JPH3, KBTBD13, KCNA1, KCNC3,KCND3, KCNE3, KCNJ10, KCNJ18, KIAA0196, KIF1A,KIF1B, KIF1C, KIF5A, KLHL40, KLHL41, L1CAM, LAMA1,LAMA2, LARGE, LDB3, LITAF, LMNA, LPIN1,LRSAM1, MARS, MARS2, MATR3, MED25, MFN2,MPZ, MRE11A, MTM1, MTMR14, MTMR2, MTPAP,MTTP, MUSK, MYF6, MYH2, MYH7, MYOT, NDRG1,NEB, NEFL, NGF, NIPA1, NOP56, NTRK1, OPA1, OPA3,OPHN1, PABPN1, PANK2, PDK3, PDYN, PEX7, PFKM,PGAM2, PHKA1, PHYH, PLEC, PLEKHG5, PLP1, PMM2,PMP22, PNKP, PNPLA6, POLG, POLG2, POMGNT1,POMT1, POMT2, PRKCG, PRPS1, PRX, PTF1A, PTRF,PYGM, RAB7A, RAPSN, REEP1, RNF216, RRM2B,RTN2, RUBCN, RYR1, RYR2, SACS, SBF2, SCN4A,SCN9A, SEPN1, SETX, SGCA, SGCB, SGCD, SGCE,SGCG, SH3TC2, SIL1, SLC12A6, SLC16A2, SLC1A3,SLC33A1, SLC39A4, SLC52A2, SLC9A1, SLC9A6, SMN1,SNX14, SOD1, SPAST, SPG11, SPG20, SPG21, SPG7,SPTBN2, SPTLC1, SPTLC2, STAC3, STUB1, SUCLA2,SYNE1, SYNE2, SYT14, TBP, TCAP, TDP1, TECPR2,TGM6, TK2, TMEM240, TNNI2, TNNT1, TPM2, TPM3,TPP1, TRIM32, TRPV4, TTBK2, TTN, TTPA, TTR,TUBB4A, TYMP, VAMP1, VCP, VLDLR, VPS13A,VPS37A, VRK1, WFS1, WNK1, WWOX, XK, YARS,ZFYVE26, ZFYVE27, ZNF592
